# Monoclonal IgA Antibodies for Aflatoxin Immunoassays

**DOI:** 10.3390/toxins8050148

**Published:** 2016-05-12

**Authors:** Özlem Ertekin, Şerife Şeyda Pirinçci, Selma Öztürk

**Affiliations:** 1 Genetic Engineering and Biotechnology Institute, Marmara Research Center, The Scientific and Technological Research Council of Turkey, TÜBİTAK, Gebze, Kocaeli 41400, Turkey; ozlem.ertekin@tubitak.gov.tr (O.E.); seyda.pirincci@tubitak.gov.tr (S.S.P.); 2 Department of Molecular Biology and Genetics, Graduate School of Sciences, Gebze Technical University, Gebze, Kocaeli 41400, Turkey; 3 Department of Medical Genetics and Molecular Biology, Kocaeli University, Umuttepe, Kocaeli 41100, Turkey

**Keywords:** mycotoxin, monoclonal antibody, immunoglobulin A, orientation, immunoaffinitycolumn, ELISA

## Abstract

Antibody based techniques are widely used for the detection of aflatoxins which are potent toxins with a high rate of occurrence in many crops. We developed a murine monoclonal antibody of immunoglobulin A (IgA) isotype with a strong binding affinity to aflatoxin B1 (AFB1), aflatoxin B2 (AFB2), aflatoxin G1 (AFG1), aflatoxin G2 (AFG2) and aflatoxin M1 (AFM1). The antibody was effectively used in immunoaffinity column (IAC) and ELISA kit development. The performance of the IACs was compatible with AOAC performance standards for affinity columns (Test Method: AOAC 991.31). The total binding capacity of the IACs containing our antibody was 111 ng, 70 ng, 114 ng and 73 ng for AFB1, AFB2, and AFG1 andAFG2, respectively. Furthermore, the recovery rates of 5 ng of each AF derivative loaded to the IACs were determined as 104.9%, 82.4%, 85.5% and 70.7% for AFB1, AFB2, AFG1 and AFG2, respectively. As for the ELISA kit developed using non-oriented, purified IgA antibody, we observed a detection range of 2–50 µg/L with 40 min total test time. The monoclonal antibody developed in this research is hitherto the first presentation of quadruple antigen binding IgA monoclonal antibodies in mycotoxin analysis and also the first study of their utilization in ELISA and IACs. IgA antibodies are valuable alternatives for immunoassay development, in terms of both sensitivity and ease of preparation, since they do not require any orientation effort.

## 1. Introduction

Aflatoxins (AFs) are hepatotoxic mycotoxins that are produced by *Aspergillus* spp. Aflatoxin B1 (AFB1), aflatoxin B2 (AFB2), aflatoxin G1 (AFG1) and aflatoxin G2 (AFG2) are the four common naturally occurring analogues of AF and AFM1 is found in milk as a product of animal metabolism of AFB1. High level exposure to AFs results in acute toxicity which may lead to death, and chronic exposure often leads to liver diseases including liver cancer in humans [1,2,3].

AF levels in food and feedstuff is regulated in many countries due to their toxic effects [4,5] and several methods are devised to fulfill the requirements of the regulations such as liquid chromatography based methods including high pressure liquid chromatography (HPLC) or liquid chromatography-tandem mass spectrometry (LC-MS/MS); and enzyme based immunological test methods including enzyme linked immunosorbent assay (ELISA) [2,6]. Instrumental AF analysis requires a series of steps including sampling, homogenization, extraction, extract clean-up and detection. The extract clean-up step comprises affinity based purification techniques, particularly immunoaffinity chromatography in order to concentrate and remove the AFs from complex extract matrix [7]. Both immunoaffinity chromatography and ELISA systems utilize the ability of an anti-AF antibody to specifically bind AFs.

There are five major classes of antibodies secreted from the B cells of mammalian systems; immunoglobulin M (IgM), immunoglobulin G (IgG), immunoglobulin A (IgA), immunoglobulin D (IgD) and immunoglobulin E (IgE). Antibodies of IgG isotype are the most abundant immunoglobulin class in mammals and accordingly, they are the most commonly developed monoclonal antibodies. Monoclonal IgGs are preferred in detection systems with their high affinities and abundance. High affinity of IgG antibodies is the result of affinity maturation which is a process where B cells produce antibodies with increased affinity to the antigen. IgM and IgD antibodies are usually not affinity maturated and hence, generally low affinity, where other antibody isotypes are affinity maturated and have higher affinities to the antigen. These antibodies also differ in valency where IgG, IgD and IgE are divalent, IgA is tetravalent and IgM is decavalent [8]. IgA isotype is a multivalent antibody which experiences affinity maturation towards the antigen. Despite potential high affinity and four antigen binding sites of IgA antibodies make them good candidates, there are limited number of reports regarding their use in immunodetection and diagnostics [9,10]. Previously known anti-AF antibodies are mostly IgG isotype antibodies [7,11], where a limited number of studies utilize IgM class of antibodies [12]. However, no IgA class monoclonal anti-mycotoxin antibody has been reported to be used in analytical systems to date.

The structural character of the antibody, which is determined by its isotype, is important for the antibody immobilization strategy. Antibody orientation is critical while immobilizing divalent antibodies to a support since random immobilization causes activity loss due to hindrance of variable regions which should be exposed in order to be functional [13]. When IgG antibodies are randomly immobilized to a solid support, about 50% of the antibody activity is impaired [14]. This results in the need for purified antibody solutions for concentrated binding to the immunosorbent surface, a higher amount of the immunosorbent material and/or a means of orienting the antibody, so that the antigen binding sites are free. The use of multivalent antibodies may be a solution to this problem as there will be free antigen binding sites even when some are blocked during immobilization.

The objective of the present work was to develop novel murine monoclonal antibody of IgA class which do not necessitate the orientation prior to immobilization on an immunosorbent surface and show its use in ELISA and immunoaffinity column (IAC) development.

## 2. Results and Discussion

### 2.1. Antibody Development

Monoclonal IgA antibodies were developed with hybridoma method [15] using AFB1-human apo transferrin (hTF) conjugate immunized Balb/C mice. The fusion yielded 878 hybrid clones, 31 of which produced positive results in direct ELISA test. Nineteen of the responsive clones showed cross-reactivity with hTF, ovalbumin (OVA) and Bovine Serum Albumin (BSA), the proteins used in immunization and ELISA tests. Twelve clones which do not react with proteins were tested for their interaction with soluble mycotoxins. Ochratoxin A (OTA), zearelanone (ZEA) and Fumonisin B1 (FB1) which poses as other risk factors in the food and feedstuff were used to test antibody cross reactivity besides AF derivatives. Five of the tested clones were inhibited by AF derivatives but not with OTA, ZEA or FB1. Three of the specific clones failed to continue antibody production with consecutive passages. The remaining two clones were tested for antibody isotypes, and the clone MAM-D12E2 producing IgA isotype antibodies were sub cloned to monoclonal cell line. The cloning and selection phase was repeated 3 times until stable hybridoma cell line MAM-D12E2 was obtained.

IgAs can be present in a monomeric and a dimeric isoform [16,17]. Dimeric structure of the MAM-D12E2 antibody was confirmed with SDS PAGE and western blot analysis (Supplementary Figure S1).

**Figure 1:**
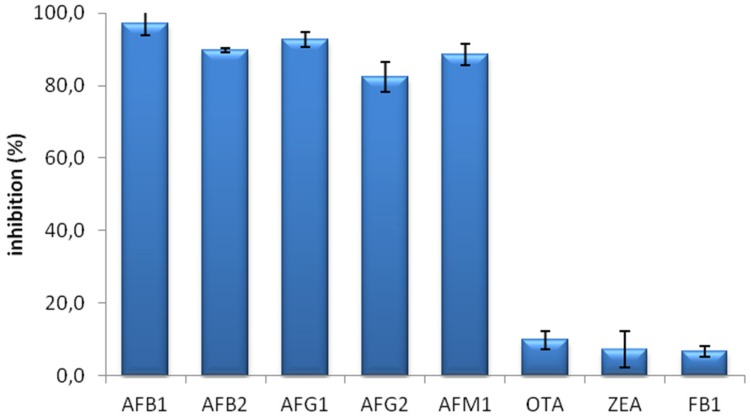
Mycotoxin inhibition assay showing the specificity of D12E2 antibody. D12E2 antibody was inhibited by all four natural analogs of AF; aflatoxin B1 (AFB1), aflatoxin B2 (AFB2), aflatoxin G1 (AFG1), aflatoxin G2 (AFG2) and the water soluble metabolite aflatoxin M1 (AFM1). Error bars represent standard deviation (SD) from three independent replicates.

The specificity of the MAM-D12E2 antibody was assessed with mycotoxin inhibition assay (Figure 1). The antibody showed strong binding to AFs, including AFM1 and was not inhibited by OTA, ZEA or FB1.

### 2.2. Development of IAC with MAM-D12E2 Antibody

IACs were prepared with MAM-D12E2 antibodies pre-concentrated with ammonium sulphate (AS) precipitation with no further purification. Each IAC was prepared by conjugating 110 µg antibody containing AS precipitate to 400 µL CNBr activated sepharose.

In antibody purification procedures, antibodies are exposed to extreme pH values, salt concentrations or heat, which may result in aggregation and loss of activity. Loss of active molecules during purification is also a strong possibility. Moreover, the use of unpurified antibodies in immunoanalytical techniques not only saves the activity, but also saves time and cost. So, being able to use the antibody sans-purification is a big advantage. Purification is necessary when high antibody concentration is required on a surface. In IgG antibodies, almost half of the antibody activity is lost due to wrong orientation [14]. The solution to this problem is either orienting the antibodies so that their paratopes are exposed, or using highly purified antibodies to be able to compensate the loss with higher antibody density. MAM-D12E2 IACs were prepared without the need for orienting and further purifying the antibody.

The resulting IACs were evaluated with two parameters; AF binding capacity and the ability to bind limited amount of AFs.

**Figure 2:**
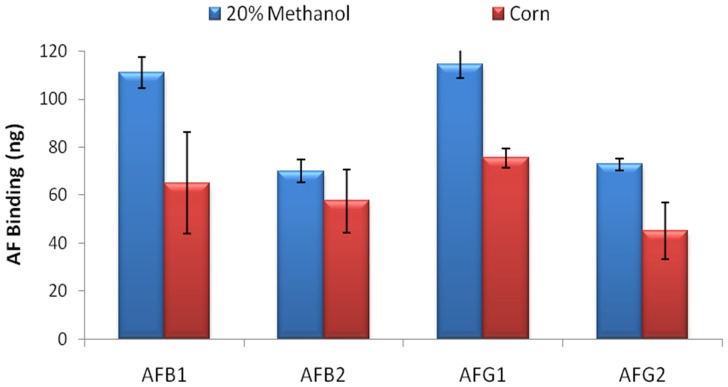
Total AF binding capacity of D12E2 immunoaffinity columns (IACs) upon 500 ng toxin loading. IACs can bind 111 ng AFB1, 70 ng AFB2, 114 ng FG1 and 73 ng AFG2 in 20% methanol-water solution. Error bars represent standard deviations.

AF binding capacity of the columns was evaluated by overflow test. 500 ng of AFB1, AFB2, AFG1 and AFG2 in 20 mL 20% methanol-water were loaded to the columns in separate experiments and total binding capacity of the columns were calculated by HPLC analysis for each AF derivative. The overflow test results showed that the IACs prepared by partially purified MAM-D12E2 antibody were able to bind 111 ± 6.5 ng AFB1, 70 ± 4.9 ng AFB2, 114 ± 5.8 ng AFG1 and 73 ± 2.4 ng AFG2 (Figure 2) where errors represent standard deviations. The test was repeated with corn extract. 34%–41% reduction in total AF binding capacity was observed for AFs B1, G1 and G2 and 18% reduction was observed for AFB2 (Figure 2).

**Figure 3:**
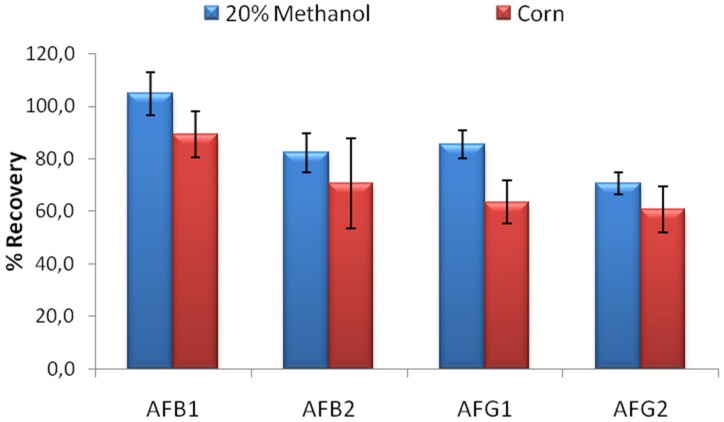
Limit detection test performed in 20% Methanol and Corn extract. A quantity of 5 ng of each AFB1, AFB2, AFG1 and AFG2 was loaded to the IAC developed with D12E2 antibody and the recovery rates were calculated after HPLC analysis.

The limit binding test is critical to evaluate the actual performance of the columns. In food extracts, the expected toxin concentration is generally 5–10 µg/L where different regulations apply for different countries [18]. The developed column should be able to bind to at least 80% of 5 ng AFB1, AFB2 and AFG1; and 60% of 5 ng AFG2 according to AOAC performance standards for affinity columns [19] in order to be used effectively in food analysis. Limit binding test for IACs developed using MAM-D12E2 antibody was conducted using 20% methanol-water solution contaminated with a mixture of AFB1, AFB2, AFG1 and AFG2 so that 5 ng of each toxin was loaded to the columns. The percentage of the recovered AFs was used for evaluation. The limit binding test results showed that the recovery rates observed with IACs prepared by partially purified MAM-D12E2 antibody were 104.9% for AFB1, 82.4% for AFB2, 85.5% for AFG1 and 70.7% for AFG2. Experiments conducted with corn extracts denoted the effectiveness of the columns for AF analysis in corn (Figure 3).

**Figure 4:**
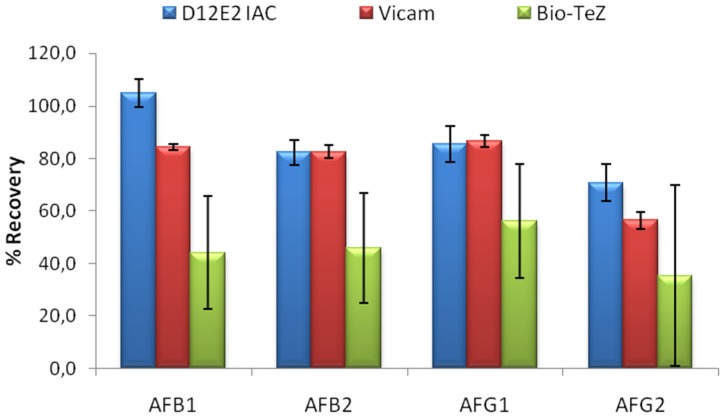
Limit detection test performed with loading 5 ng of each AFB1, AFB2, AFG1 and AFG2 to the IAC developed with D12E2 antibody and two commercial AF IACs. Error bars represent standard deviations.

The performance of MAM-D12E2 IAC was compared to two commercial IACs; Vicam and BioTeZ. The isotypes of the antibodies used in these IACs was shown to be IgG by using isotype specific AP labeled secondary antibodies (Supplementary Table S1).In this experimental setup, 16% methanol concentration was chosen to accord the test conditions to the requirements of the commercial IACs. 20 mL 16% methanol was contaminated with 5 ng of each AF derivative. Comparative evaluation of limit binding performances of the IACs is presented in Figure 4. The results showed the superior performance of MAM-D12E2 IAC in terms of AF binding performance and better repeatibility when compared to BioTeZcolumns.

### 2.3. Development of ELISA Test with MAM-D12E2 Antibody

ELISA test system based on direct competitive ELISA for rapid, easy and on-site detection and quantification of AFB1 was developed by the use of purified MAM-D12E2 antibody. The designed test system involved the immobilization of MAM-D12E2 antibody to the ELISA plate without any need for orientation. Quantification was based on the competition of enzyme labeled AFB1-protein conjugate with different concentrations of AF in the solution. AFB1-protein conjugate was labeled with HRP with the use of gluteraldehyde method [20]. The test enabled the quantification of AFB1 in corn and hazelnut extracts.

**Figure 5:**
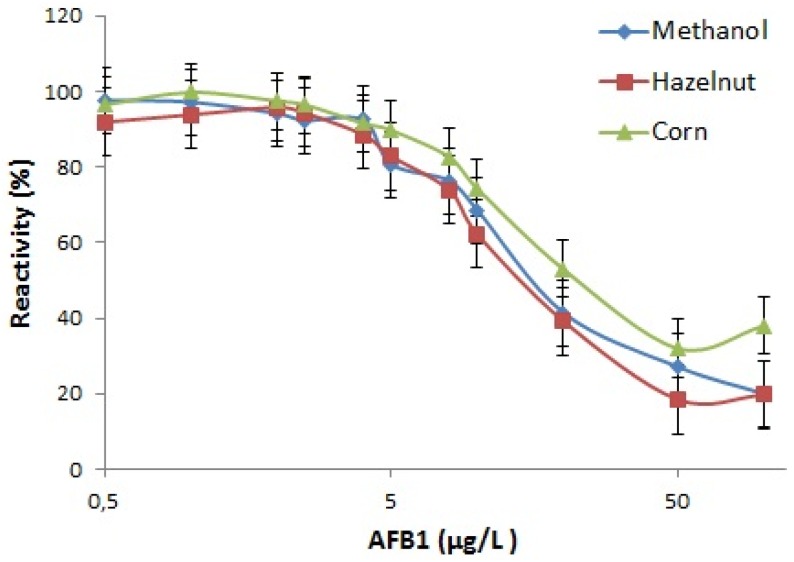
AFB1 inhibition curve of the ELISA test developed with D12E2 antibody in 17.5% methanol and in the presence of Hazelnut and Corn extracts with 17.5% final methanol concentration. The error bars represent standard error.

Utilization of IgA as coating antibody is expected to provide higher number of free antigen binding sites in the ELISA wells, which will potentially lead to broader range of detection. Detection range of the kit in methanol was determined as 2–100 µg/L and in AFB1 spiked extracts was 2–50 µg/L. Detection between these limits allows control of all foods and feeds for AFB1 contamination except baby foods and cereals intended for infants which requires detection below 0.1 µg/L [4]. Maximal absorbance of control samples with no AFB1 competition, IC50 and IC10 values for methanol, corn and hazelnut standard curves in 17.5% final methanol concentration are presented in Table 1. The sensitivity of the kit was correlated with control methanol solution in corn and nut extracts. Calibration curves in 17.5% methanol and in hazelnut and corn extracts are shown in Figure 5.

Despite the developed ELISA test system was not optimized for commercial production, the test was able to detect AFB1 in broader concentration range than its commercial counterparts (Sigma, St. Louis, MO, USA; *SE120001*, 0.2–4 µg/L and *SE120002* 0.02–4 µg/L; Helica, Santa Ana, CA, USA, 941BAFL01-96 and 981BAFL01LM-96, 1–20 µg/L) which is considered as an advantage of IgA antibody.

Although the standard curve of the ELISA test was constructed using AFB1 derivative as standard toxin, the kit can easily be adopted to total AF quantification. MAM-D12E2 antibody has high affinity to AFB2, AFG1 and AFG2 as well as AFB1. In a sample contaminated with more than one of the derivatives, individual quantification of AF derivatives would require IAC purification followed by HPLC analysis.

## 3. Materials and Methods

### 3.1. Materials

We used all chemical reagents from Sigma Aldrich (St. Louis, MO, USA) with the exception of 1-Ethyl-3-(3-dimethylaminopropyl) carbodiimide (EDC) (Thermo Scientific, Waltham, MA, USA) and AFs B1, B2, G1, G2, M1, OTA, ZEA and FB1 (Fermentek, Jerusalem, Israel).

### 3.2. Preparation of AF-Protein Conjugates

The AFB1 conjugates include AFB1-hTF and AFB1-OVA which were utilized as AF immunogen in mice immunizations, and AFB1-BSA which was utilized as the solid phase antigen to be used in indirect and competitive indirect ELISA tests.

The carboxyl groups of the protein carrier was first cross-linked to ethylenediamine (EDA) with 1-Ethyl-3-[3-dimethylaminopropyl]-carbodiimide hydrochloride (EDC) linker so that the amine groups of the protein were enriched with the method previously described [21]. The unreacted EDA and EDC were removed by dialysis against distilled water.

AF-protein conjugation was achieved by Mannich type reaction. The amine groups of cationized proteins were condensed with formaldehyde and the α-hydrogen adjacent carbonyl in AFB1 [22]. AFB1-protein conjugate was purified by washing the conjugate with 0.1 M MES, pH: 4.8 MES at least five times by 10 kDa MWCO centrifugal filters. AF-protein conjugates were stored in small aliquots at −20°C.

### 3.3. Immunization

Five Balb/c mice were intraperitonally immunized with 100 µg of AFB1-hTF conjugates. In the first dose of injection, complete Freund’s adjuvant was used. The subsequent doses were prepared with incomplete Freund’s adjuvant. The mice were bled at day 12 after each immunization and sera were collected by centrifugation. Anti-AF antibody titer was evaluated with 10.000 fold PBS diluted sera by indirect ELISA with 500 ng AFB1-BSA coated wells. In order to ensure that antibodies were capable of binding free AFs, mycotoxin inhibition assay with AFB1 was performed. Five doses of the conjugate were injected to mice until the desired antibody response was achieved. A booster immunization with 50 µg of AFB1-OVA conjugate was intravenously injected to the mouse with highest AF specific antibody response. Animal experiments were performed in compliance with the appropriate laws and institutional guidelines with the approval of TÜBİTAK Marmara Research Center, Genetic Engineering and Biotechnology Institute ethical committee.

### 3.4. Cell Fusion and Selection

Monoclonal antibodies were developed with hybridoma technique as explained elsewhere [23]. Murine myeloma cell line F0 was used for the hybridoma production. Spleen cells of the mouse which received the booster immunization and F0 cells were mixed with 1:3 F0:spleen cell ratio. The fusion was achieved by the addition of 1 mL 50% PEG 4000 solution to the cells at 37°C. Fusion clones were selected with Hypoxanthine-Aminopterine-Thymidine (HAT) medium.

The immortalized cells were screened and selected for their anti-AF antibody production performance by indirect ELISA with 500 ng AFB1-BSA coated plates. Supernatants of the antibody producing hybridoma clones were tested for their cross reactivity with the proteins used in immunizations by indirect ELISA. Clones producing antibodies tested negative for protein cross reactivity were further evaluated for AF specificity by mycotoxin inhibition assay. AF specific antibody producing clones were selected and antibody isotypes were determined with mouse monoclonal subisotyping kit (BD biosciences, San Jose, CA, USA). Among clones producing AF specific antibodies, IgA isotype antibody producing clone was sub cloned to a monoclonal cell line.

### 3.5. Antibody Production and Handling of the Monoclones

Monoclonal antibodies were obtained from the cell culture supernatant of the selected hybridoma cell line. The stable IgA producing hybridoma cells were grown in 5% fetal bovine serum (FBS) containing Dulbecco’s Modified Eagle Medium (DMEM). Cells were frozen in 10% DMSO, 20%FBS and 70% DMEM containing medium. Antibody was purified with anion exchange chromatography with DEAE Cellulose column by modified standard protocols [24] after ammonium sulphate (AS) precipitation.

### 3.6. Mycotoxin Inhibition Assay

ELISA plates were coated with 500 ng AFB1-BSA conjugate and blocked with 1% skimmed milk solution. 1 µg of different mycotoxins (AFs B1, B2, G1, G2, M1, OTA, ZEA and FB1) were preincubated with 1/2 diluted cell culture supernatant for 30 min. at 37°C. 2000 fold PBS diluted Alkaline phosphatase (AP) conjugated rabbit anti mouse polyvalent antibody was used as secondary antibody. AP substrate 4-nitrophenyl phosphate was dissolved in substrate buffer (1 mM ZnCl_2_, 1 mM MgCl2, 0.1M glycine, pH 10.4) with 1 mg/mL concentration. Absorbance at 405 nm was measured with a microplate reader.

### 3.7. Indirect ELISA

500 ng AFB1-BSA was coated to the ELISA plates. Some wells were incubated with PBS as negative control. The wells were blocked with 1% skimmed milk solution in PBS. 10,000 fold diluted mice sera, undiluted culture supernatant or 1000 fold diluted antibody AS precipitate were dispensed to the designated wells, depending on the test conducted. Non-immunized mouse sera or PBS were used as negative controls. 2.000 fold diluted AP conjugated rabbit anti mouse polyvalent antibody was used as secondary antibody. 4-nitrophenyl phosphate was used as AP substrate. Absorbance at 405 nm was measured with a microplate reader.

### 3.8. Immunoaffinity Column Preparation

CNBr activated sepharose 4B (Sigma C9142) was activated in acidic pH. AS precipitate of the D12E2 antibody was 10 fold diluted with binding buffer (0.1 M NaHCO, 0.5 M NaCl, pH 8.3). 1 mL of diluted antibody solution was used to bind 1 mL sepharose. The unbound moieties on the sepharose were blocked with 1 M Ethanolamine, pH: 8. 400 µL resin was used per column.

### 3.9. Evaluation of Immunoaffinity Columns

Column performance was evaluated with overflow and limit binding tests. 

For the overflow test 20% methanol-water solution or 20% methanol containing corn extract which was used as model matrix were contaminated with 25 µg/L AF B1, B2, G1 or G2. For the limit binding test, similarly prepared methanol and corn extracts were contaminated with a mixture of 0.25 µg/L AFB1, AFB2, AFG1 and AFG2. For comparative analysis of in-house prepared AF IACs with two different commercial IACs, limit binding test was performed with similarly contaminated 16% methanol-water solution. 20 mL of the samples were loaded to the columns at 3 mL/min flow rate and the columns were washed with 20 mL distilled water. The bound AF was eluted with 1 mL methanol. The limit binding test was conducted in comparison with two different commercial IACs; BioTeZ (BioTeZIAC Aflatoxin; Size 3 mL; BTAF310005, Berlin, Germany) and Vicam (AflaTest columns, G1010, Milford, MA, USA). The D12E2 IAC tests were conducted with 10 replicates. BioTeZIAC was used with 5 replicates and Vicam IAC was used with 2 replicates.

AF concentrations in eluates were evaluated by HPLC analysis. CoBrA cell method was employed for the derivatization of the AFs. 20 µL sample was loaded to 250/4.6 Nucleosil 100-5 C18 column (Macherey-Nagel 720014.46) at 1 mL/min flow rate at room temperature. 55% KBr-HNO_3_ buffer (238 mg/L KBr and 700 µL 4M HNO_3_/L) + 27% methanol + 18% acetonitrile was used as mobile phase. Toxins were detected with Fluorescence detector at 360 nm excitation and 430 nm emission wavelength. Device was calibrated with certified AF standards for accurate quantification.

### 3.10. Preparation of ELISA Test System

AFB1-BSA was conjugated to horse radish peroxidase (HRP) using gluteraldehyde method [20]. 700 ng of purified antibody was coated to each ELISA plate well in PBS by overnight incubation at +4°C. The wells were blocked with 1% skimmed milk solution in PBS. Corn and hazelnut extracts prepared according to the AOAC regulations [19] were used as model matrices in ELISA test system.400 ng AFB1-BSA-HRP conjugate was mixed with varying concentrations of unconjugated AF in 70% methanol-water solution or 70% methanol containing food extracts in 3:1 ratio to a final methanol concentration of 17.5%. PBS is used for dilution of the extracts. Competition with clean extract was used as negative control. Every sample/standard was used twice. After 20 min of incubation at room temperature, wells were washed five times with 0.2% Tween-PBS. TMB HRP substrate was added to the wells and incubated for 20 min. The reaction was stopped after incubation with 2 M H_2_SO_4_. Absorbance at 450 nm was measured with a microplate reader. The standard curve was plotted by using average inhibition values (%).

### 3.11. Safety Considerations

AFs are known to be hepatotoxic and AFB1 is a class 1 human carcinogen. Therefore, all experiments were conducted at a biosafety level 2 laboratory with proper protection.

## 4. Conclusions

This work is hitherto the first application of IgA isotype antibodies in detection systems. The applicability of IgA monoclonals in immunoanalytical systems was demonstrated with an AF specific murine monoclonal IgA antibody, MAM-D12E2. ELISA kit and IACs fulfilling the requirements of international standards were developed with the presented antibody. Immobilization of antibodies to the support matrix of IACs and ELISA plates were achieved without any need for antibody orientation, which was discussed as the primary advantage of IgA isotype antibodies. The high affinity and specificity of the antibody allowed the application of the developed systems in food matrices. The results obtained with MAM-D12E2 IgA monoclonal antibody were superior to its IgG counterparts in IACs. Hence, IgA monoclonals were shown to be good alternatives to commonly used IgG monoclonals. Further studies are to be conducted with ELISA and Biosensor systems for the evaluation of comparative performance of IgG and IgA isotype antibodies.

## Figures and Tables

**Table 1:** Maximal absorbance, IC50 and IC10 values of ELISA test in 17.5% final methanol concentration.

Parameter	Methanol	Hazelnut	Corn
OD 450 * (maximal)	1.41±0.05	1.26 ± 0.02	1.2 ± 0.02
IC10 ** (µg/L)	28.19	25.11	32.38
IC50 *** (µg/L)	54.21	48.90	60.23

* Maximal absorbance of negative control at 450 nm; ** 50% inhibitory concentration; *** 10% inhibitory concentration.

## Supplementary Materials

The following are available online at www.mdpi.com/2072-6651/8/5/148/s1, Figure S1: SDS PAGE and Western blot analysis (**A**); Silver staining (**B**). Immunoblot analysis. M: SDS PAGE marker (Sigma MWND500), Lane 1: Purified D12E2; Lane 2: Purified control IgG, Table S1: Isotype determination of commercial IACs. Optical densities at 405 nm are presented.

## Abbreviations

The following abbreviations are used in this manuscript:

AF	Aflatoxin
AFB1-OVA	Aflatoxin B1 and Ovalbumin Conjugate
AFB1-BSA	Aflatoxin B1 and Bovine Serum Albumin Conjugate
AFB1-hTF	Aflatoxin B1 and Human Apo transferrin Conjugate
AP	Alkaline Phosphatase
AS	Ammonium Sulphate
BSA	Bovine Serum Albumin
DMEM	Dulbecco’s Modified Eagle Medium
DMSO	Dimethyl Sulphoxide
EDC	1-Ethyl-3-[3-dimethylaminopropyl]-carbodiimide hydrochloride
ELISA	Enzyme Linked Immunosorbent Assay
FB1	Fumonisin B1
FBS	Fetal Bovine Serum
HPLC	High Performance Liquid Chromatography
HRP	Horse Radish Peroxidase
hTF	Human Apo transferrin
IAC	Immunoaffinity Column
MES	2-(*N*-morpholino)-ethane sulfonic acid
OTA	Ochratoxin A
OVA	Ovalbumin
PBS	Phosphate-Buffered Saline
PEG	Polyethylene glycol
ZEA	Zearelanone
