# Supplementary material for: Monoclonal IgA Antibodies for Aflatoxin Immunoassays
Source: Toxins (Basel). 2016 May 12;8(5):148. doi: 10.3390/toxins8050148 (PMC4885063; doi:10.3390/toxins8050148)
Supplement: Supplementary file 1 [file toxins-08-00148-s001.pdf]

Supplementary Materials: Monoclonal IgA Antibodies for Aflatoxin Immunoassays

Özlem Ertekin, Şerife Şeyda Pirinçci and Selma Öztürk

Antibody Valency Determination

Materials and methods

The valency of D12E2 Mab was assessed by Sodium Dodecyl Sulfate Polyacrylamide Gel Electrophoresis (SDS-PAGE) and western blotting. A monoclonal IgG antibody was used as control. For SDS-PAGE analysis, 2 μg of purified antibodies were mixed with 4×Laemmli Sample Buffer under non-denaturing conditions. Sigma Aldrich Kit for MW 14.000–500.000 (Sigma-Aldrich, St. Louis, MO, USA, MWND500) was used as molecular weight marker. Proteins were loaded on discontinuous gel system with 5% (*w*/*v*) polyacrylamide loading gel and 12% (*w*/*v*) separating gel. Electrophoresis was conducted with Bio-Rad mini protean tetra cell gel apparatus under 120 V continuous voltage. Gels were stained with silver staining. For Western blot analysis, the proteins on the gels were transferred on a PVDF membrane in methanol containing transfer buffer (0.02 M Trizma base, 0.15 M Glycin, 0.0003% SDS and 20% Methanol) using a semi-dry Western blot apparatus (Bio-Rad, Hercules, CA, USA, Trans-Blot Turbo Transfer System) for 30 min at 200 mA. Membrane was then blocked with 1% skim milk powder. 1:5000 diluted AP labeled Rabbit anti mouse polyvalent antibody was incubated with the membrane for 1 h. The blots were visualized on the membrane with AP substrate (Sigma-Aldrich, St. Louis, MO, USA, B5655).

Results

D12E2 and control IgG were analyzed by SDS PAGE and western blot which is followed by an immunoblot aiming to visualize the antibodies transferred to the membrane. In the SDS PAGE and Western blot analysis, 2 μg protein was loaded from purified antibodies. Analysis was done in non-denaturing conditions so that antibody subunits will not be separated and antibodies could be observed in theirintact multimeric forms. SDS PAGE analysis presented in Figure S1A shows the exact same amount of protein that were transferred to the PVDF membrane for immunoblot analysis shown in Figure S1B. Both SDS PAGE analysis and western blot shows higher molecular weight of D12E2 antibody with respect to control IgG and no bands were observed at 150 kDa indicating there was no observable monomeric IgA in the antibody solution.

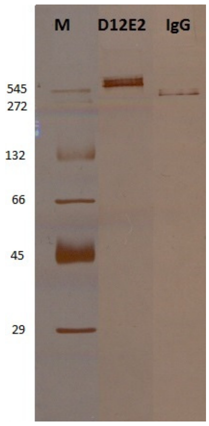

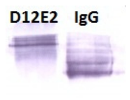

(**A**) (**B**)

Figure S1. SDS PAGE and Western blot analysis (**A**); Silver staining (**B**). Immunoblot analysis. M: SDS PAGE marker (Sigma MWND500), Lane 1: Purified D12E2; Lane 2: Purified control IgG.

Antibody Isotyping for Commercial IACs

Materials and methods

Commercial Bio TeZ and VicamIACs were analyzed for antibody isotypes. 300 µL column resin from each IAC was separated to 75 µL aliquots. Aliquots were washed with 500 µL 0.2% Tween-PBS buffer with centrifugation for 3 times. Each of four aliquots were treated with 2.000 fold diluted AP labeled Anti-mouse polyvalent, IgG, IgM or IgA antibodies in PBS and washed with PBST as stated earlier. 500 µL 1 mg/mL 4-nitrophenyl phosphate was used as AP substrate. Absorbance at 405 nm was measured with a spectrophotometer.

Results

Antibody isotypes used in commercial IACs BioTeZ and Vicam were determined by using AP conjugated isotype specific secondary antibodies. The results obtained from the test are presented in **Table S1**. Positive response was observed by the interaction of the resin with anti-mouse polyvalent (IgG, IgA, IgM) antibody and anti-mouse IgG antibody.

Table S1. Isotype determination of commercial IACs. Optical densities at 405 nm are presented.

Labeled Antibody	Bio TeZ	Vicam
Anti-mouse Polyvalent	2.219	2.076
Anti-mouse IgG	2.394	2.197
Anti-mouse IgM	0.183	0.236
Anti-mouse IgA	0.237	0.347
